# Active Video Games Improve Muscular Fitness and Motor Skills in Children with Overweight or Obesity

**DOI:** 10.3390/ijerph19052642

**Published:** 2022-02-24

**Authors:** Cristina Comeras-Chueca, Lorena Villalba-Heredia, Jose Luis Perez-Lasierra, Jorge Marín-Puyalto, Gabriel Lozano-Berges, Ángel Matute-Llorente, Germán Vicente-Rodríguez, Alex Gonzalez-Aguero, José A. Casajús

**Affiliations:** 1 Faculty of Health and Sport Science (FCSD), Department of Physiatry and Nursing, Universidad de Zaragoza, 50009 Zaragoza, Spain; ccomeras@unizar.es (C.C.-C.); glozano@unizar.es (G.L.-B.); amatute@unizar.es (Á.M.-L.); gervicen@unizar.es (G.V.-R.); alexgonz@unizar.es (A.G.-A.); 2 GENUD (Growth, Exercise, NUtrition and Development) Research Group, Department of Physiatry and Nursing, Universidad de Zaragoza, 50009 Zaragoza, Spain; lvillalbaheredia@unizar.es (L.V.-H.); jlperez@unizar.es (J.L.P.-L.); jmarinp@unizar.es (J.M.-P.); 3 EXERNET Red de Investigación en Ejercicio Físico y Salud, 50009 Zaragoza, Spain; 4 Faculty of Health Science, Department of Physiatry and Nursing, Universidad de Zaragoza, 50009 Zaragoza, Spain

**Keywords:** exergaming, muscular fitness, motor competence, exercise, children, obesity

## Abstract

(1) Background: Childhood obesity is an important public health problem. Children with overweight or obesity often tend to show the pediatric inactivity triad components; these involve exercise deficit disorder, pediatric dynapenia, and physical illiteracy. The aim of the study was to examine the influence of an active video games (AVG) intervention combined with multicomponent exercise on muscular fitness, physical activity (PA), and motor skills in children with overweight or obesity. (2) Methods: A total of 29 (13 girls) children (10.07 ± 0.84 years) with overweight or obesity were randomly allocated in the intervention group (AVG group; *n* = 21) or in the control group (CG; *n* = 8). The intervention group performed a 5-month AVG training using the Xbox 360^®^ with the Kinect, the Nintendo Wii^®^, dance mats, and the BKOOL^®^ interactive cycling simulator, combined with multicomponent exercise, performing three sessions per week. The control group continued their daily activities without modification. Weight, PA using accelerometers, and motor competence using the Test of Gross Motor Development 3^rd^ edition were measured. Muscular fitness was evaluated through the Counter Movement Jump height, maximal isometric strength of knee extension and handgrip strength, and lean mass using Dual-energy X-ray Absorptiometry. Mann–Whitney U and Wilcoxon signed rank tests were performed. The biserial correlation coefficients (r) were calculated. Spearman’s correlation coefficients among PA, muscular fitness, and motor competence variables were also calculated. (3) Results: The AVG group significantly increased their knee extension maximal isometric strength (4.22 kg; *p* < 0.01), handgrip strength (1.93 kg; *p* < 0.01), and jump height (1.60 cm; *p* < 0.01), while the control group only increased the knee extension maximal isometric strength (3.15 kg; *p* < 0.01). The AVG group improved motor competence and light physical activity (*p* < 0.05) and decreased sedentary time (*p* < 0.05). Lean mass improved in both AVG group and CG (*p* < 0.05). Lastly, the percentage of improvement of motor skills positively correlated with the percentage of improvement in vigorous PA (r = 0.673; *p* = 0.003) and the percentage of improvement in CMJ (r = 0.466; *p* = 0.039). (4) Conclusions: A 5-month intervention combining AVG with multicomponent training seems to have positive effects on muscle fitness, motor competence, and PA in children with overweight or obesity.

## 1. Introduction

Obesity has become a major global health challenge because of its increased prevalence, which has risen significantly over the past three decades [1]. The World Health Organization (WHO) even refers to obesity as “a global pandemic” [2]. Childhood obesity is considered an important public health problem worldwide [3]. The prevalence of overweight and obesity among children and adolescents (aged 5–19) has dramatically risen from 4% in 1975 to just over 18% in 2016 worldwide [4]. The prevalence of obesity in children and adolescents in Spain between the ages of 8 and 16 (the stage of life in which young people are most susceptible to excess body fat) reached 34.9% in 2019 [5]. These results are in line with European results that showed a prevalence of overweight over 30% and a prevalence of obesity over 10% in European children and adolescents in 2016 [6].

Both overweight and obesity are associated with adverse health consequences such as cardiovascular, respiratory, and metabolic diseases; negative psycho-social effects; and are more likely to become overweight or obese adults, resulting in an increased risk of non-communicable diseases [7].

An important factor of childhood obesity is insufficient physical activity (PA). According to the WHO, children and adolescents should participate in an average of 60 min per day of moderate-to-vigorous intensity physical activity (MVPA) across the week, and could expect additional benefits from a greater amount of PA [8]. Nevertheless, a large proportion of children and adolescents do not meet the public health recommendations of PA, with over 80% of the world’s adolescent population being insufficiently physically active in 2016 [9].

Physical inactivity is related to the pediatric inactivity triad, a condition observed by Faigenbaum et al. [10] that involves exercise deficit disorder, pediatric dynapenia, and physical illiteracy. The term “exercise deficit disorder” is used to highlight the gravity of this clinical condition with the aim of raising awareness of the importance of meeting the public health recommendations of PA. Children with pediatric dynapenia show low levels of muscle strength and power and, consequently, functional limitations that are not caused by neurological or muscular diseases [10]. The concept of “physical illiteracy” describes the lack of competence, confidence, and motivation to move proficiently [10].

The deficit of PA is closely related to other components since children and adolescents who show lack of muscular fitness and a decreased level of motor skills are less likely to participate in sport activities or just PA, making it impossible to improve muscular fitness or motor skills [10]. PA is a powerful tool to improve youth health [11] and is favorably associated with the management of adiposity, cardio-metabolic biomarkers, health-related physical fitness (including muscular fitness), bone health [12,13], psychological health, motor competence, and quality of life [14].

The importance of muscular fitness lies in its inverse association with total and central adiposity and therefore with cardiovascular diseases and metabolic risk factors, and its positive association with bone health [15] and self-esteem [16]. Children and adolescents with overweight and obesity show higher maximal muscular strength than children and adolescents with normal weight because the excess of weight involves a chronic training stimulus during the activities of daily living [17]. In contrast, when maximal muscular strength is normalized to body mass or lean mass, those with overweight are weaker, probably as a result of reduced mobility, neural adaptations, and changes in muscle morphology [18,19]. Therefore, children and adolescents with a higher weight status had negative effects on the performance of strength tests that involve moving their body weight, showing e.g., a lower jump height [20]. However, children and adolescents with a higher weight status had positive effects on performance of strength tests that do not involve lifting the body such as isometric knee extension strength or handgrip test [20]. Likewise, overweight and obesity are associated with deficient muscular strength and endurance [21,22]. Similarly, children with overweight or obesity showed poorer muscle tissue composition, lower muscle power, and alterations in motor unit recruitment, which led to impaired muscle activation [23]. To this should be added the current declining trend in muscular fitness and the need to target those strength deficits in today’s youth [24]. Given the above, children and adolescents with overweight or obesity are more likely to fulfil the pediatric dynapenia condition of the pediatric inactivity triad (PIT) [10].

Lastly, children and adolescents with overweight and obesity are often less skilled than those with healthy weight [25]. Actual and, especially, perceived motor skills are associated with motivation, PA, and sports participation in children and adolescents [26,27], so the improvement of motor skills within this population must be a main goal. In addition to the positive relationship with PA, motor competence has been positively related positively to cardiorespiratory fitness [28] and inversely related to sedentary time, overweight, and obesity [29].

Active video games (AVG) have been proposed as a good alternative for traditional exercise and have become an emerging trend in fitness [30], being investigated to find out its effectiveness against childhood obesity and in promoting PA and health in children and adolescents [31]. AVG are video games that require body movement and therefore an increase in energy expenditure [31,32]. The strategy is to partner with the digital movement in the world to seek to increase PA and achieve health benefits through AVG rather than fighting technology. AVG can be useful tools for improving PA, health-related physical fitness, and motor skills, however, it seems that using AVG exclusively does not provide the benefits needed [33,34,35,36]. Therefore, AVG must be used as a complementary activity in the fight against childhood obesity [34,35,37,38,39]. Evidence supports the use of a structured AVG intervention instead of home-use, with stronger effects of a multicomponent exercise intervention combined with AVG [40].

In addition, AVG seem to have positive effects on motor skills and health-related physical fitness. Some articles have shown improvements in the acquisition and development of motor skills and abilities in a funnier way for the children [41]. Improvements in health-related physical fitness have been shown after an AVG intervention, such as cardiorespiratory and muscular fitness [42] and body composition [43].

However, to the best of our knowledge, no randomized controlled trials (RCT) have been performed to confirm the benefits of AVG in muscular fitness, PA, and motor skills altogether. The effect of an intervention with AVG on the PIT has not yet been investigated, despite being key in the children of today. In addition, an AVG intervention combined with multicomponent exercise could be a novel way of increasing the effects of AVG.

Therefore, the main aim of the study was to examine the influence of an AVG intervention combined with multicomponent exercise on muscular fitness, PA, and motor skills in children with overweight or obesity.

The authors hypothesized that AVG could help to improve the isometric strength of upper and lower limbs and the height jump in a vertical jump, as well as to increase the lean mass, motor skills, and PA without resorting to more traditional sports, which do not seem to be a sufficiently attractive option for overweight or obese children and adolescents.

## 2. Materials and Methods

This study was performed in accordance with the ethical guidelines for human research outlined by the Declaration of Helsinki (revision of Fortaleza 2013) [44] and the Declaration of Taipei [45], and were reviewed and approved by the Research Ethics Committee of the Government of Aragon (certificate No. 11/2018, CEICA, Zaragoza, Spain). All participants and their parents or legal custodians were informed of the nature and possible risks of the experimental procedures before their written informed consent was obtained.

This RCT is part of a larger cross-over study that is registered in clinicaltrials.gov (identification number NCT04418713). In this RCT, participants were divided into two groups, an intervention group that participated in an AVG exercise program combined with multicomponent exercise (*n* = 21), and a control group (*n* = 8) that continued their daily activities without modification.

The recruitment process was carried out through pediatricians from the medical centers. Informative talks about the activity were given in medical centers and it was the pediatricians themselves who proposed the activity to overweight or obese patients who could benefit from the activity. Due to the difficulty of this recruitment process, it was planned to extend the study for one more year in order to obtain a bigger sample. Therefore, a 2:1 randomization was carried out in the first year, prioritizing the AVG intervention. The Statistical Package for the Social Sciences (SPSS) version 23.0 (SPSS Inc., Chicago, IL, USA) was used to generate the random allocation sequence, and it was performed by the researcher who carried out the project. In a second moment, randomization was 2:1, favoring the control group. Unfortunately, it was interrupted by the COVID-19 pandemic and groups were, in the end, uneven. The second part of the study with a new recruitment process was interrupted by the pandemic caused by COVID-19.

### 2.1. Participants

A total of 29 children with overweight or obesity met the inclusion criteria and participated in the RCT. There were a total of 16 boys and 13 girls. The inclusion criteria were as follows: the participants had to be between the ages of 9 and 12 years, in Tanner stage I or II, and not having had menarche, with overweight or obesity established following the cut points of Cole et al. [46]. Tanner’s stage was evaluated by a medical doctor by direct observation. Volunteers suffering from pathologies that worsen with physical exercise or having any other contraindications for its practice were excluded from the present study. In addition, the exclusion criteria were the following: participating in regular high-level or high-intensity extracurricular PA, following any special dietary regime, and taking any medication that may interfere with the evaluated variables.

The participants were recruited from medical centers through their pediatricians of Zaragoza (Spain). The involvement of pediatricians in recruitment is especially important because overweight and obesity are health conditions to be treated, so they refer those children to us. Parents and pediatricians were informed about the development of the activity, the results, and the progress of the children.

### 2.2. Outcomes

#### 2.2.1. Anthropometry

All participants were measured with a stadiometer without shoes and minimum clothing to the nearest 0.1 cm (SECA 225, SECA, Hamburg, Germany) and weighed to the nearest 0.1 kg (SECA 861, SECA, Hamburg, Germany). The body mass index (BMI) was calculated as weight (in kg) divided by height (in m) squared.

#### 2.2.2. Physical Activity

PA and sedentary behaviors were objectively assessed before and after the intervention using the calidated GENEActiv accelerometers (ActivInsights Ltd., Kimbolton, Cambridgeshire, UK) [47]. All participants wore the accelerometer on their left wrist for 7 consecutive days [48]. Devices were set to record at a frequency of 30 Hz.

The instruction was to wear the devices during the whole day for 7 days, including during sleep and water-based activities. Verbal instructions were given to the parents along with a timesheet to register in case the device was removed.

The accelerometer data were downloaded using GENEActiv software 3.2 version and aggregated into 15-s epochs for its posterior analysis using R software. Non-wear periods were detected following the method proposed by van Hees et al. [49] and eliminated from the registers. After that, participants with less than 4 valid days (≥10 h of wear time per day) were excluded from the accelerometry analysis. Time during sedentary behaviors and at different PA intensities (light (LPA), moderate (MPA) or vigorous (VPA)) was determined according to the cut-off points proposed by Schaefer et al. [50], namely 0.190 g, 0.314 g, and 0.998 g for light, moderate, and vigorous intensities, respectively. The sleep time was not included in sedentary time. The total PA was calculated as the sum of minutes in light, moderate, and vigorous intensities.

#### 2.2.3. Pediatric Dynapenia

Pediatric dynapenia was evaluated through three tests: Counter Movement Jump (CMJ) height, maximal isometric strength of knee extension test, and handgrip strength test. Both weight-related and non-weight-related tests were used to assess the muscular strength in upper and lower limbs. In addition, lean mass was assessed as part of pediatric dynapenia.

##### Counter Movement Jump Height

CMJ was evaluated using a Kistler force plate 9260AA (Kistler Holding AG, Winterthur, Switzerland). Children were in a standing position with both hands on their hips to isolate the lower-limb action. For the performance of the countermovement, children were asked to go down fast and not to stop after going down. Three attempts were permitted, and the best performance was recorded for further analysis. Normalized scores were obtained based on the results of the study performed by Focke et al. [51]. The jump height was calculated from the flight time using the following equation: $h_{t}=\frac{g{\left(t_{v}\right)}^{2}}{8}$ [52].

##### Maximal Isometric Strength

Maximal isometric strength of knee extension was measured by a signal-frame gauge (Universidad de Zaragoza, Zaragoza, Spain). Children started from a sitting position with their knees flexed 90°. An anchorage was placed on the anterior distal third of the tibia. This anchorage was connected to the strain gauge, registering force data during the time that the participant had to perform the maximum knee extension until the force curve began to decrease. Three attempts were permitted for each leg, with the best performance recorded from each leg. The mean of the best attempt for each leg was obtained to quantify isometric knee extension strength. Normalized scores were obtained based on the reference values of the study performed by Beenakker et al. [53].

##### Handgrip Strength

Handgrip strength test was measured using a handgrip dynamometer (TKK 5001, grip A; Takei) (range, 0–100 kg; accuracy, 0.1 kg). Children were in a standing position maintaining the arm of the tested side straight down with the shoulder slightly abducted (~10° not touching the rest of the body), the elbow in 0° of flexion, the forearm in neutral position, and the wrist in 0° of flexion. The best value of three attempts with each hand was used. Handgrip strength percentiles by age and sex were calculated based on normative values of children and adolescents aged 9–17 years representing 24 countries proposed by Tomkinson et al. [54].

##### Lean Mass

Dual energy X-ray absorptiometry scans were performed with the pediatric version of the QDR-Explorer software (Hologic Corp., Software version 12.6.1, Bedford, MA, USA) for the whole body (and its sub-regions). Subtotal (total body less head), legs (calculated as a mean of both legs), and arms (calculated as a mean of both arms) lean mass were obtained from whole body scans. All DXA analyses were performed by the same trained researcher. The coefficient of variation of DXA in the laboratory for lean mass was 1.9% [55]. Furthermore, lean mass index normalized to height was calculated and normalized scores were obtained based on the reference values of the study performed by Weber et al. [56].

#### 2.2.4. Motor Skills

Motor skills were assessed in the AVG group, using the Test for Gross Motor Development—3rd Edition (TGMD-3), which assesses 13 fundamental motor skills, subdivided into locomotor and object control domains [57]. TGMD-3 was tested and coded according to the standardized administration and grading. A detailed explanation of administration and coding can be found in the web page of TGMD-3 [58], with general information, a brief administration guideline, some helpful videos, and four reliability videos. Researchers who evaluated the motor skills with this tool completed the four reliability videos evaluation to check the consistency of administration and coding and the intra- and inter-observer variability. The score of tests, result coding, and percentiles were obtained based on the examiner’s manual [59].

### 2.3. Intervention

The AVG group was engaged in a 5-month intervention with three sessions per week and 60 min per session that included a combination of AVG and multicomponent training. The sessions were composed of 10 min of warm-up, including: joint mobility, dynamic flexibility, muscle activation, core, balance, and coordination exercises. This was followed by the main part, which consisted of 45 min of exercise with a combination of AVG and multicomponent exercises, following a dynamic circuit workout where the participants were continuously rotating from AVG to exercises, and finally a 5 min cool-down performing static flexibility routines. The multicomponent exercise was focused on health-related physical fitness including cardiorespiratory fitness, muscular strength, agility, and coordination.

The AVG and the multi-component training exercises were carried out according to the plan. Two out of the three weekly sessions performed by the participants aimed at improving cardiorespiratory fitness and one at improving muscular fitness. One of the sessions focused on cardiorespiratory fitness included a muscle strength part. The exercises were multi-articular and functional, combining upper and lower body exercises, and aimed to complement the main part using the AVG. Balance, coordination, and agility work was included in the last part of the warm-up to be done without fatigue.

A progression in difficulty of performance and intensity was followed both in the AVG part and in the multi-component training. Each AVG started at the simplest and lowest intensity and ended with the most complex and highest intensity. As for the exercises in multicomponent training, initially low-difficulty and lower-intensity exercises were performed with the aim of inducing learning and self-awareness, and evolving as this learning took place. Thus, an intensity close to 3 MET was planned for the first month, in the following 3 months the intensity was intended to be between 3 and 6 MET, and in the last month it was intended to exceed 6 MET. Taking all sessions into account, the average intensity was 5.4 ± 1.1 MET.

In general, the sessions consisted of four AVG with a total average duration of 24 min, and the multicomponent exercise was performed between the AVG. The multicomponent exercise lasted 13 min on average per session, divided into two or three activities with different aims depending on the planning.

In the main part, the AVG included were the following: The Xbox 360^®^ with the Kinect using “Kinect Adventures” and “Kinect Sport”; the Nintendo Wii^®^ using “Wii Sports”, “Just Dance”, and “Mario and Sonic at the Olympic Games”; dance mats using “Dance Dance Revolution” and “Mario and Sonic at the Olympic Games” adapted from the Nintendo Wii to the dance mats; and the BKOOL^®^ interactive cycling simulator connected to a tablet HUAWEI MediaPad T5 AGS2-W09.

The intervention was carried out in two locations, the University of Zaragoza and the “San Braulio” public school in Zaragoza. The sessions were different every day, following a progression of difficulty and intensity and fulfilling the objectives previously established in the planning.

### 2.4. Statistical Analyses

The Statistical Package for the Social Sciences (SPSS) version 23.0 (SPSS Inc., Chicago, IL, USA) was used to perform all the statistical analyses. Statistical significance was set at *p* < 0.05 in all tests. Data are presented as mean ± standard deviation (SD). Kolmogorov–Smirnov tests were performed to verify the normal distribution of the variables. As several variables did not show a normal distribution, non-parametric tests were performed.

Differences between AVG and control groups for descriptive characteristics, muscular fitness, physical activity, and motor skills at the pre- and post-intervention were analyses using Mann–Whitney U tests. Wilcoxon signed-rank test was used for within-group comparisons among the pre-intervention and post-intervention measures. Effect size r was calculated for Mann–Whitney U and Wilcoxon signed-rank tests using the formula of Fritz, Morris and Richler [60] for non-parametric contrast/comparisons. The effect size for r can be small (0.1–0.3), medium (0.3–0.5), or large (>0.5) [61]. Spearman’s correlation coefficients among the improvement and the percentage of improvement for PA, muscular fitness, and motor competence variables were calculated.

## 3. Results

Descriptive characteristics of participants by group are summarized in Table 1. No differences between groups at baseline or post-intervention were found (r ranged from 0.082 to 0.335; *p* > 0.05).

**Figure 1:**
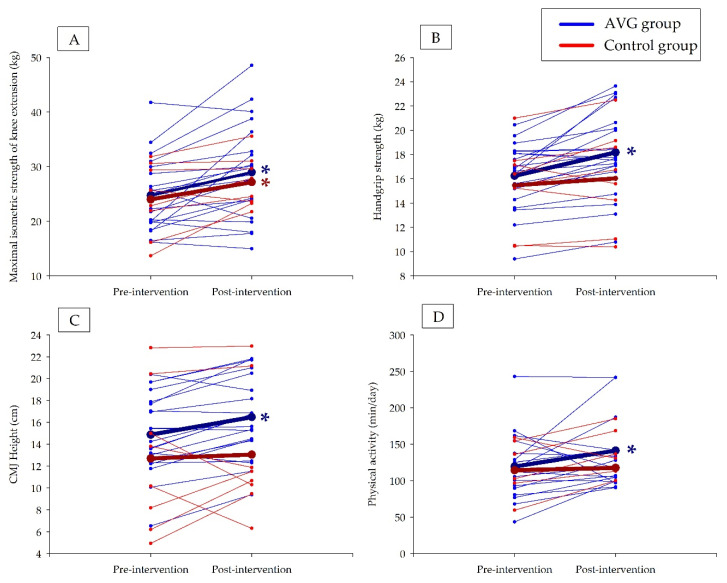
Individual changes for muscular fitness and physical activity variables in AVG and control groups. AVG: active video games; CMJ: counter movement jump. The thickest line represents the mean. * Significant changes between pre-intervention and post-intervention in AVG group were set at *p* < 0.05. (**A**) Maximal isometric strength of knee extension interactions in the AVG group and the control group. (**B**) Handgrip strength interactions in the AVG group and the control group. (**C**) CMJ height interactions in the AVG group and the control group. (**D**) Physical activity per day interactions in the AVG group and the control group.

Muscular fitness in the AVG group and the control group is shown in Table 2. Both the AVG group and the control group significantly increased subtotal lean mass, index, and Z-Score of indexes of subtotal lean mass and lean mass of arms and legs (*p* < 0.01; Table 2). The AVG group significantly increased the knee extension maximal isometric strength (*p* < 0.01; Figure 1A), hand-grip test (*p* < 0.01; Figure 1B), and CMJ together with z-score of CMJ test (*p* < 0.01 and *p* < 0.05 respectively; Figure 1C). Handgrip significantly improved in the AVG group but not in the control group when it was corrected by body weight. The control group increased maximal knee extension isometric strength (*p* < 0.01; Figure 1A). It is worthy to highlight that a significant increase in maximal knee extension isometric strength was found in the AVG group, whereas no changes were found in the control group when the isometric strength of quadriceps was corrected by body weight.

PA in both AVG and control group is shown in Table 3. Regarding physical inactivity, in the AVG group 93.8% of the participants did not meet the public health recommendations of PA before the intervention, lowering to 75.0% afterwards. In the control group, all the participants were insufficiently active, reducing to 85.7% after the intervention. It should be noted that some data losses occurred due to a failure in one accelerometer register, which prevented the data recorded by the accelerometer from being obtained. It should be noted that some PA data could not be used due to malfunctioning of the device, so data were obtained from 16 children in the AVG group and 7 children in the control group, and only those were analyzed overall. A significant reduction of sedentary time was observed in the AVG group (*p* = 0.034), whereas no significant changes were observed in the control group. LPA significantly increased in the AVG group (*p* = 0.02), whereas no significant changes in MPA and VPA were shown in either the AVG group or the control group. An increase in total daily PA was shown in the AVG group while no effects were shown in the control group (Figure 1D). No group by time interactions were found in PA and muscular fitness variables (*p* > 0.05; Table 2 and Table 3).

**Figure 2:**
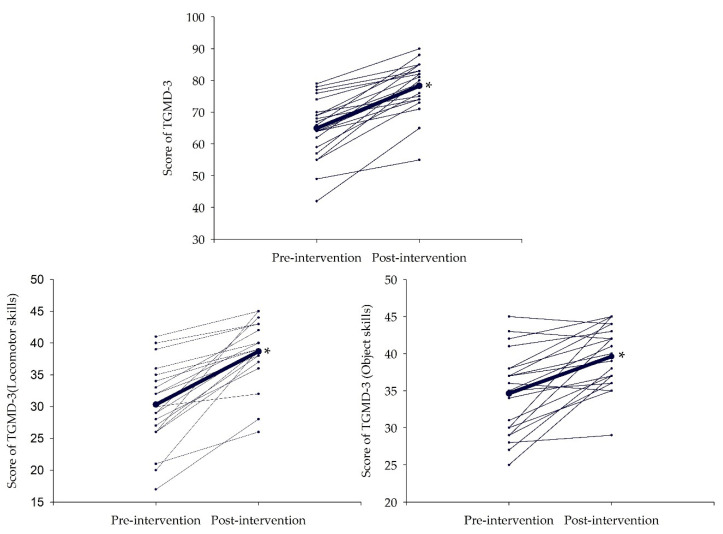
Individual changes for the score of motor competence of the AVG group. TGMD-3: Test of gross motor development 3rd edition. The thickest line represents the mean. The unit of measurement is points. * Significant interactions were set at *p* < 0.05.

As can be seen in Table 4, the AVG group improved motor skills in both the locomotive skills and the object skills (all *p* < 0.001; Figure 2). According to the scores obtained in TGMD-3, the AVG group was in the 5th percentile before the intervention, and had an improvement after it, up to the 22nd percentile.

All participants assigned to the intervention group attended a minimum of 80% of the sessions, indicating high adherence to the program and at the end of the program, 100% of the participants were willing to continue with the program.

Lastly, correlations were observed between the percentage of improvement of motor skills and the percentage of increment in CMJ (r = 0.466; *p* = 0.039) and between the percentage of improvement of motor skills and the percentage of increment in VPA (r = 0.663; *p* = 0.004). Also, some relations between improvement of muscle fitness and the improvement of PA were found. Specifically, the results showed that the percentage of improvement of Z-Score of lean mass index was correlated with the percentage of improvement of VPA (r = 0.434; *p* = 0.039). The improvement and the percentage of improvement of handgrip strength test was positively correlated with the increase of arm lean mass (r = 0.492; *p* = 0.007; and r = 0.459; *p* = 0.012) and with the increase of total lean (r = 0.463; *p* = 0.012), subtotal lean (r = 0.421; *p* = 0.023), and Z-Score of index of lean mass (r = 0.463; *p* = 0.012).

## 4. Discussion

The main finding of this study is that this AVG intervention has the potential for improving muscular fitness in overweight and obese children, and can have some impact on motor skills related variables. Results showed that whereas both groups increased lean mass as they are growing populations, only AVG group experienced a significant increase in CMJ test and handgrip strength tests. In addition, both groups increased isometric knee extension strength, possibly due to the development of lean mass as a consequence of maturational development. However, significant improvements in isometric knee extension strength were only found in the AVG group when the results were corrected by body weight, which means that the gain of the isometric knee extension strength was not entirely due to the increase in body weight, unlike the control group whose significant increases disappeared when dividing by body weight. Therefore, as the authors hypothesized, AVG seem to be an effective tool to improve the muscular fitness and to increase PA and motor skills in children with overweight or obesity, according to the results of this article. The high attendance of the participants assigned to the AVG group and the willingness and desire to continue with the exercise program through active video games showed a high motivation to participate.

Up to now, only three non-controlled trials [62,63,64] have investigated the effects of an AVG intervention on muscular fitness of children and adolescents with overweight and obesity. Calcaterra et al. [62] showed an increase in muscular strength of 22 obese children, improving from 29.6 ± 9.3 to 32.3 ± 9.8 kg (*p* = 0.003) in handgrip test. Duman et al. [63] investigated the effects of an AVG intervention combined with traditional exercise on several physical performance tests that require muscle strength and endurance, such as time to ascend and descend 20 stairs, number of squats they can perform in 120 s, time to run 50 m, and number of rope jumps in 30 s. The results showed enhancements in all test performances. Lastly, Huang et al. [64] investigated the effect of AVG on muscular strength. No changes in muscular strength of the quadriceps or muscular endurance were found. Low frequency and program duration may explain the lack of significant changes.

Participants in this study showed a high maximal muscular strength of knee extension (Z-Score) and a high performance in the hand-grip test (percentile), probably due to the chronic stimulus produced by their weight excess, while they showed lower values of jump height in both the initial and the final measurements, with results according to the reference values [51]. Therefore children with overweight or obesity are more likely to fulfil the pediatric dynapenia condition of the pediatric inactivity triad [10], so it seems necessary to bet for strength training also in the interventions with overweight and obese children. AVG could be an interesting and promising strategy to improve muscular fitness and fight against the pediatric dynapenia of overweight and obese children.

Both the AVG and control groups significantly increased subtotal lean mass and lean mass of arms and legs. Since there are no significant differences between the AVG group and the control group, it seems that these increases can be the result of growth and development rather than the intervention. Furthermore, in both groups, the index of subtotal lean mass increased, with a higher increase in the AVG group, and the Z-Score of the index of subtotal lean mass decreased, with a higher decrease in the control group, even showing a negative Z-Score value. The AVG intervention does not appear to influence this variable. Despite this, there seems to be a positive trend in the lean mass index and lean mass index Z-Score variables favoring the AVG group. However, issues such as the lack of dietary control or the progression of intensity during the intervention that resulted in not reaching as high intensities as necessary makes it difficult to detect significant changes. Another explanation for the lack of differences in lean mass after AVG intervention may be that there is insufficient muscle stimulation. On the other hand, other interesting AVG devices such as the Nintendo Switch Ring Fit Adventures, which incorporates a muscle resistance implement, might provide higher muscle stimulation. It would be interesting to study the effect of the Ring Fit Adventures, especially considering the restrictions on physical exercise due to COVID-19, with home exercise predominating.

To the best of our knowledge, there are four controlled trials that informed about or reported the effects of AVG on lean mass [65,66,67,68]. Maddison et al. [65] did not find changes between groups for lean mass. Similar results were reported by Adamo et al. [66], with no differences between groups. Staiano et al. [67,68] informed about no effects on lean mass after an AVG intervention. Evidence on the effect of AVG on lean mass is limited and no effects were shown. All the articles investigating the effects of AVG on lean mass [65,66,67,68] lasted less than 6 months. This could explain why no positive effects compared to control groups were observed. Thus, interventions lasting 6 months or more may be needed to observe clear benefits compared to a control group, also taking into account growth and muscle development in the pre-pubertal stages. In addition, the previously mentioned articles were mainly focused on the decrease of body fat and not so much on the increase of lean mass. However, in the present study, multicomponent training focused on the improvement of muscular strength, which was included as one of the objectives.

Total PA and LPA significantly increased in the AVG group (*p* < 0.01). No significant changes in MPA and VPA were shown in either the AVG group or the control group. However, the AVG group showed an increasing trend in MPA and VPA. The importance of this significant increase in LPA lies in the opportunity to replace sedentary time with LPA [69], and in this way, to fight against the opposite trend of replacing LPA with more sedentary time that occurs in pre-adolescence and adolescence [70]. In addition, LPA has a strong influence on cardio-metabolic health [69]. In addition, the AVG group showed significant reductions in sedentary time which, together with the LPA results, implies a very positive effect of the AVG intervention.

A very up-to-date systematic review performed by Gao et al. [36] concluded that AVG appears to be a promising and attractive strategy to promote PA in children with overweight or obesity. Other meta-analyses reported that AVG could be a good alternative for sedentary behavior and could be complementary to traditional exercise [34,71]. Mack et al. [35] agree with the use of the AVG as a complementary tool to increase PA, claiming that the exclusive use of AVG does not achieve satisfactory results. AVG could promote light-to-moderate intensity PA and replace sedentary screen-time [72], but the long-term benefits in PA are limited [73]. AVG could even be an exercising strategy to help meet PA recommendations [74]. In short, AVG could help achieve a more active and healthy lifestyle among children with overweight and obesity [33], provided that the AVG interventions were supervised and structured [40]. On the other hand, several systematic reviews do not support the effectiveness of AVG to increase PA levels sufficiently [38,39,75]. This ineffectiveness may be due to the wrong approach to AVG interventions in the studies included in these reviews, carried out without supervision, with an insufficient length or with insufficient participants. On the other hand, several systematic reviews [34,35,37,71] stress that AVG can be a complementary tool to increase PA and health, so multicomponent training has been included in the intervention of this study in a novel way.

A noteworthy improvement in the motor competence of the AVG group was observed. The lack of data on the motor competence of the control group due to time, material, and available evaluator is a major limitation, which made a comparison with a control group impossible. Nevertheless, the improvement in the motor competence of the AVG group is quite considerable, so it hardly seems to be explained by children’s growth and development alone. In accordance with the results of the present study, Van Biljon et al. [76] showed improvements in the AVG group in motor ability compared to two control groups, a control group with access to traditional sedentary video games and another control group that did not receive any intervention. Motor competence was evaluated in 30 overweight and obese children using the short form of the Bruininks-Oseretsky test for motor proficiency, and the AVG group performed a 6-week AVG intervention of 3 days per week for 30 min per session using Wii. The AVG group showed improvements in motor competence compared with both control groups; specifically, in agility and speed, coordination, and reaction time. Another recent article [77] also evaluated the improvement of motor skills in primary school students with overweight or obesity, with the same limitation as the present study since the results could not be compared with a control group. To evaluate the motor competence, three movements (catch, vertical jump, and kick) were analyzed and a significant improvement in the score of vertical jump movement was observed, in addition to the improvement in jumping height. A systematic review concluded that AVG, used as a complementary tool and not as the only tool, can be a good strategy to improve motor competence in children [41]. This conclusion supports the intervention performed in the present study, which combines AVG with multicomponent exercise and justifies the improvements found.

Actual and perceived motor competence are positively associated with motivation for exercising, PA, and sports participation in children and adolescents, and inversely associated with sedentary time, overweight, and obesity [26,27], which is especially important in children with a high weight status [29]. According to the interrelationship of the components of the pediatric inactivity triad [10] (exercise deficit disorder, pediatric dynapenia, and physical illiteracy), an increase of motor competence could lead to an increase of PA, which in turn could lead to an improvement in muscle fitness and motor competence. This interrelationship can also happen in a reverse way, so that a worsening of the motor skill can lead to a deficit of PA that produces a worsening of the muscular fitness and of the motor skill. This process is the same in children who do not develop their motor skills in a normal way and show a worse motor competence, which usually happens in children who are overweight or obese [25]. In addition, this physical illiteracy that usually appears in children with a high weight status increases over time, with all the difficulties that it entails in terms of adherence to exercise and improvement of the health-related physical fitness, including muscular fitness.

The pediatric inactivity triad is a novel conceptual approach that reflects a public health crisis in today’s children and adolescents. Despite the forcefulness of this concept, which expresses the tendency of children to be weaker, less agile, slower, less active, and more overweight, there are currently no articles focused on proposing solutions or strategies to this reality. Furthermore, although this concept is defined, no cut-off points or normative data have been identified to determine whether the pediatric inactivity triad, namely pediatric dynapenia and physical illiteracy, is met. A great contribution to future research would be the possibility of detecting this pediatric triad of inactivity in the pediatrician’s office through simple tests that could be carried out in the office itself.

Lastly, the present study found correlations between the percentage of improvement of motor skills and the percentage of improvement in VPA. This relationship suggests that children with better motor skills can reach higher intensities. The pediatric inactivity triad supports this interrelationship between these two variables that together with muscular fitness make it up. The key to this condition lies in the influence of its components, explaining that a deficit of PA will lead to a worsening of motor skills in children and to muscular weakness, which in turn will lead to a lower motivation to perform PA and a decrease in it. The correlation between motor competence and VPA is supported by Barnett et al. [78], showing a positive association between PA and motor competence and coordination, but also showing an inverse relationship between sedentary time and motor competence. There are more articles supporting this association between motor competence and PA [79,80], stating that motor competence may be a key determinant of PA behaviors across time and primary school years are the optimal time to develop motor competence [80]. A positive association between motor competence and muscular fitness was expected since the development of motor competence in childhood may improve health-related physical fitness, according to Cattuzzo et al. [81]. Stricker et al. [82] highlighted the importance of strength in children and adolescents and its relationship to motor competence. A very current study shows potential positive effects for the integrated combination of fundamental movement skill and strength training when delivered in a primary school setting with the aim of improving motor competence [83]. On the other hand, as expected, a correlation was found between the percentage of improvement of motor skills and the percentage of improvement in CMJ. This can be explained by the motor skill component found in a countermovement jump, even without using the arms [84]. This result indicates that caution should be exercised when using the CMJ as a test for measuring lower body strength, given that it has a significant motor skill component that challenges leg strength. This is even more noticeable in children who are overweight or obese as they have to move their own body weight [20]. However, no association between motor competence and isometric strength was found in the results of the current study. It could be due to their higher absolute strength in the maximal isometric strength of lower and upper limbs.

There was another correlation between the percentage of improvement of Z-Score of lean mass index and the percentage of improvement of VPA, thus corroborating the importance of VPA for muscular development.

Finally, and as expected, the results showed that the improvement and the percentage of improvement of handgrip strength test was positively correlated with the increase of arm lean mass and with the increase of total lean, subtotal lean, and Z-Score of index of lean mass. This relationship between strength test performance and lean mass was expected as the tests measured upper body isometric strength, excluding the motor skill factor. However, in the lower body isometric strength tests, no relationship with lean mass was found, which may be due to the low number of participants. A systematic review supported that PA was favorably associated with muscular fitness, as well as cardio-metabolic biomarkers, cardiorespiratory fitness, motor competence, bone health, and quality of life [14].

AVG seem to have some positive effects on the components of the pediatric triad of inactivity. However, our main objective of using AVG was to catch the attention of children who are reluctant to engage in traditional PA, so that they do so voluntarily. These AVG seek to make this PA fun and enjoyable, through a virtual environment that is more comfortable for children with this profile (i.e., obesity, low motor skills). The aim was to change the relationships of these children with PA to move from rejection to habit, and in this way achieve a more active lifestyle. This goes beyond the aims of the study, but the improvement of the components of the pediatric inactivity triad through the AVG will make these overweight or obese children feel stronger, more agile, and more skilled, so they will have more desire to move and more interest in practicing other sports.

Future research may focus on the effects of interventions with AVG combined with multicomponent training on the component of the pediatric inactivity triad due to the inverse relationship between pediatric inactivity triad and obesity, and the positive relationship between pediatric inactivity triad and health and quality of life. Despite the strength of this concept, as mentioned above, there is currently a limited amount of articles focused on proposing solutions or strategies to this reality.

## 5. Limitations and Strengths

Some limitations must be considered in this study. The number of participants was low, especially in the control group. This could make it more difficult to find effects of the intervention itself, given that in some variables trends are observed that are not statistically significant, probably due to insufficient statistical power. On the other hand, another important limitation is the absence of the control group for motor competence. Although such a considerable improvement in motor skills hardly seems to be explained by children’s growth and development alone, it is necessary to consider the absence of data for the control group for this variable.

Finally, the intervention was supervised and structured, with a frequency (three sessions per week and 60 min per session) similar or higher to other interventions that reported benefits and with a duration close to what is needed to achieve positive results (5 months). Another aspect to highlight was the combination of AVG and multicomponent training focused on cardiorespiratory fitness, muscular strength, agility, and coordination. In addition, the wide variety of AVG used should be highlighted. All these devices offer opportunities and possibilities to significantly increase energy expenditure in overweight or obese children [32].

## 6. Conclusions

A 5-month intervention of AVG combined with multicomponent training seems to have positive effects on muscle fitness and motor competence. Furthermore, this intervention seems to be a potential strategy to increase LPA and decrease sedentary time, but its ability to increase MVPA is unclear. In short, AVG combined with multicomponent exercise carried out in structured and supervised sessions could be a useful tool in improving some components of the pediatric inactivity triad in overweight or obese children.

## Figures and Tables

**Table 1:** Subject characteristics of AVG group and control group.

Variable	Time	All (*n* = 29)	AVG Group (*n* = 21)			Control Group (*n* = *8*)		
		Mean ± SD	Mean ± SD	Effect Size *r*	*p* Value	Mean ± SD	Effect Size *r*	*p* Value
Age (years)	1	10.1 ± 0.8	10.2 ± 0.8	0.88	<0.001	9.7 ± 0.8	0.89	0.012
	2	10.7 ± 0.8 *	10.8 ± 0.8 *			10.5 ± 0.8 *		
Weight (kg)	1	53.4 ± 8.9	55.3 ± 8.8	0.74	0.001	48.2 ± 7.2	0.84	0.017
	2	56.3 ± 9.8 *	57.7 ± 9.5 *			52.5 ± 10.3 *		
Height (cm)	1	144.7 ± 7.5	146.0 ± 6.7	0.88	<0.001	141.1 ± 8.8	0.89	0.012
	2	148.9 ± 7.4 *	149.6 ± 7.1 *			147.0 ± 8.3 *		
BMI (kg/m^2^)	1	25.4 ± 2.7	25.9 ± 2.9	0.15	0.498	24.1 ± 1.7	<0.00	0.990
	2	25.3 ± 3.0	25.7 ± 3.0			24.1 ± 2.8		
BMI Z-Score	1	1.96 ± 0.33	1.99 ± 0.36	0.71	0.001	1.89 ± 0.18	0.54	0.123
	2	1.84 ± 0.38 *	1.89 ± 0.41			1.72 ± 0.28		
BMI percentile	1	96.8 ± 2.1	96.9 ± 2.35	0.42	0.053	96.6 ± 1.4	0.59	0.093
	2	95.7 ± 4.0 *	95.9 ± 4.5			95.1 ± 2.5		

AVG: active video games, BMI: body mass index, SD: standard deviation. 1: pre-intervention; 2: post-intervention. Effect size r can be small (>0.1), medium (>0.3), or large (>0.5). * Significant differences within groups between pre-intervention and post-intervention (*p* < 0.05). Data presented as mean ± standard deviation.

**Table 2:** Muscular fitness by group.

Variable	Time	AVG Group (*n* = 21)	Control Group (*n* = 8)	AVG Group			Control Group		
				Change	Effect Size *r*	*p* Value	Change	Effect Size *r*	*p* Value
		Mean ± SD	Mean ± SD						
Knee extension maximal isometric strength (kg)	1	24.8 ± 6.6	24.0 ± 6.7	4.2 ± 5.7 *	0.66	0.003	3.2 ± 3.7 *	0.74	0.036
	2	29.0 ± 8.8	27.2 ± 4.7						
Knee extension maximal isometric strength relative to body weight (kg/body weight in kg)	1	0.46 ± 0.13	0.50 ± 0.12	0.05 ± 0.10 *	0.47	0.030	0.03 ± 0.11	0.15	0.674
	2	0.51 ± 0.15	0.53 ± 0.08						
Knee extension maximal isometric strength Z-Score	1	0.24 ± 1.36	0.46 ± 1.07	0.52 ± 1.38	0.39	0.073	0.14 ± 0.98	0.30	0.401
	2	0.76 ± 2.04	0.59 ± 1.17						
Knee extension maximal isometric strength Z-Score	1	0.24 ± 1.36	0.46 ± 1.07	0.52 ± 1.38	0.39	0.073	0.14 ± 0.98	0.30	0.401
	2	0.76 ± 2.04	0.59 ± 1.17						
Hand-grip strength (kg)	1	16.3 ± 2.6	15.5 ± 3.6	1.93 ± 1.93 *	0.82	<0.001	0.57 ± 1.40	0.35	0.327
	2	18.2 ± 3.4	16.0 ± 4.1						
Hand-grip strength relative to body weight (kg/body weight in kg)	1	0.30 ± 0.05	0.32 ± 0.06	0.02 ± 0.03 *	0.65	0.003	−0.02 ± 0.03	0.45	0.208
	2	0.32 ± 0.53	0.31 ± 0.05						
Percentile of hand-grip strength	1	45.5 ± 24.0	43.8 ± 19.2	3.3 ± 18.8	0.13	0.544	−5.0 ± 16.9	0.23	0.516
	2	48.8 ± 24.7	38.8 ± 23.6						
CMJ (cm)	1	14.9 ± 3.6	16.1 ± 4.6	1.6 ± 1.5 *	0.73	0.001	0.2 ± 2.5	0.10	0.779
	2	16.5 ± 3.6	16.3 ± 4.1						
CMJ Z-Score	1	−1.21 ± 0.80	−0.96 ± 0.92	0.22 ± 0.40 *	0.49	0.025	−0.01 ± 0.58	0.05	0.889
	2	−0.99 ± 0.77	−0.97 ± 0.79						
Total lean mass (kg)	1	30.7 ± 4.6	27.4 ± 5.0	2.0 ± 1.5 *	0.83	<0.001	2.3 ± 2.2 *	0.84	0.017
	2	32.7 ± 5.0	29.7 ± 6.3						
Index of subtotal lean mass	1	9.85 ± 1.99	9.15 ± 1.79	3.82 ± 1.79 *	0.88	<0.001	3.61 ± 2.15 *	0.89	0.012
	2	13.66 ± 1.40	12.76 ± 1.7						
Z-Score of index of subtotal lean mass	1	1.15 ± 0.47	1.05 ± 0.34	−0.72 ± 0.65 *	0.75	0.001	−1.11 ± 0.82 *	0.84	0.017
	2	0.44 ± 0.76	−0.06 ± 0.8						
Legs lean mass (kg)	1	5.5 ± 1.0	4.9 ± 1.0	0.4 ± 0.3 *	0.85	<0.001	0.6 ± 0.6 *	0.79	0.025
	2	5.9 ± 1.0	5.4 ± 1.4						
Arms lean mass (kg)	1	1.4 ± 0.2	1.2 ± 0.2	0.1 ± 0.1 *	0.79	<0.001	0.1 ± 0.2 *	0.79	0.025
	2	1.5 ± 0.3	1.4 ± 0.3						

AVG: active video games; CMJ: counter movement jump; SD: standard deviation, 1: pre-intervention; 2: post-intervention. Effect size r can be small (>0.1), medium (>0.3), or large (>0.5). * Significant differences within groups between pre-intervention and post-intervention (*p* < 0.05).

**Table 3:** Physical activity by group.

Variable	Time	AVG Group (*n* = 16)	Control Group (*n* = 7)	AVG Group			Control Group		
				Change	Effects Size r	*p* Value	Change	Effects Size *r*	*p* Value
		Mean ± SD	Mean ± SD						
ST (min/day)	1	841.9 ± 52.7	826.0 ± 51.2	−195.9 ± 311.2 *	0.53	0.034	−12.3 ± 268.2	0.26	0.499
	2	813.9 ± 53.6	824.2 ± 49.3						
LPA (min/day)	1	71.3 ± 20.3	77.9 ± 30.9	89.9 ± 121.4 *	0.58	0.020	−12.8 ± 154.9	0.06	0.866
	2	84.1 ± 19.2	76.1 ± 19.0						
MPA (min/day)	1	44.5 ± 22.9	35.10 ± 16.82	51.3 ± 142.2	0.44	0.079	36.6 ± 119.2	0.38	0.310
	2	51.8 ± 26.7	40.3 ± 16.6						
VPA (min/day)	1	3.9 ± 5.0	1.5 ± 1.1	9.6 ± 20.6	0.45	0.069	−0.3 ± 6.2	0.10	0.799
	2	5.3 ± 5.9	1.5 ± 1.2						
MVPA (min/day)	1	48.4 ± 27.4	36.6 ± 17.8	60.9 ± 161.2	0.47	0.063	36.3 ± 124.3	0.32	0.398
	2	57.1 ± 32.3	41.83 ± 17.7						
PA (min/day)	1	119.7 ± 43.9	122.7 ± 40.5	150.8 ± 258.5 *	0.53	0.034	23.5 ± 275.6	0.26	0.499
	2	141.2 ± 46.4	109.3 ± 33.5						

AVG: active video games; LPA: light physical activity; MPA: moderate physical activity; MVPA: moderate-to-vigorous physical activity, PA: physical activity; SD: standard deviation, ST: sedentary time; VPA: vigorous physical activity. 1: pre-intervention; 2: post-intervention. Effect size r can be small (>0.1), medium (>0.3), or large (>0.5). * Significant differences within groups between pre-intervention and post-intervention (*p* < 0.05).

**Table 4:** Motor skills in AVG group before and after the intervention.

Variable	Pre-Intervention (*n* = 21) Mean ± SD	Post-Intervention (*n* = 21) Mean ± SD	Effects Size *r*	*p* Value
Total motor skills score (points)	64.7 ± 9.7	78.3 ± 8.1 *	0.88	<0.001
Locomotor skills (points)	30.3 ± 6.5	38.7 ± 5.0 *	0.88	<0.001
Object control skills (points)	34.6 ± 5.6	39.6 ± 4.3 *	0.81	<0.001

AVG: active video games; SD: standard deviation. Effect size r can be small (>0.1), medium (>0.3), or large (>0.5). * Significant differences within groups between pre-intervention and post-intervention (*p* < 0.05).

## Data Availability

Not applicable.
